# Supplementary material for: Progerinin, an Inhibitor of Progerin, Alleviates Cardiac Abnormalities in a Model Mouse of Hutchinson–Gilford Progeria Syndrome
Source: Cells. 2023 Apr 24;12(9):1232. doi: 10.3390/cells12091232 (PMC10177486; doi:10.3390/cells12091232)

Supple Figure S1.

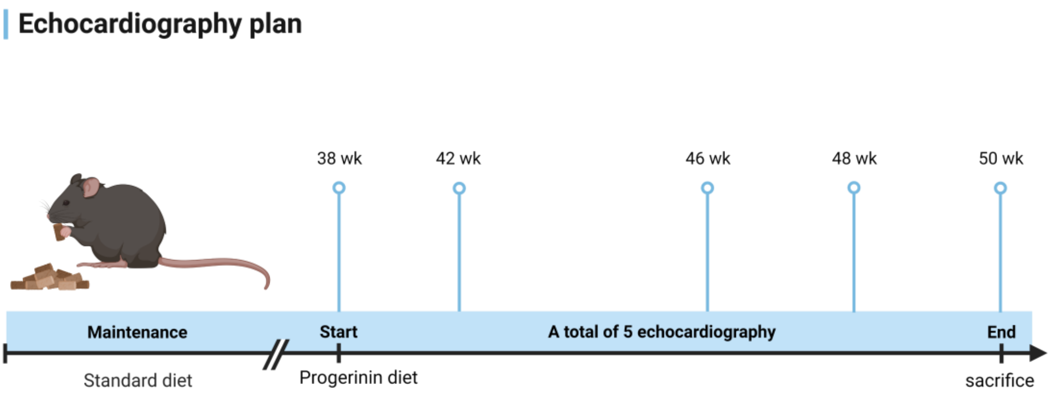

Supple Figure S1. Experimental protocol of echocardiography. Timeline of the administration of Progerinin to mice and the experimental protocols. Echocardiography was performed at 5-time points from 38 weeks to 50 weeks of age (*Lmna*$^{WT/WT}$ Vehicle: *n*=5; *Lmna*$^{WT/WT}$ Treated: *n*=6; *Lmna*$^{G609G/WT}$ Vehicle: *n*=8; *Lmna*$^{G609G/WT}$ Treated: *n*=7).

Supple Figure S2.

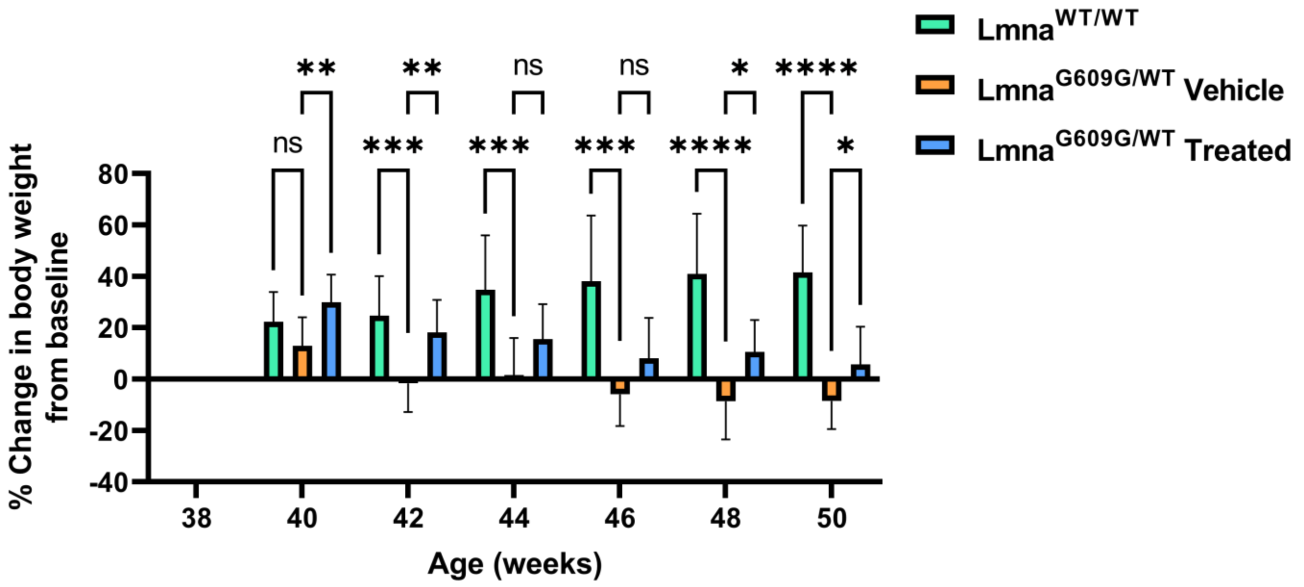

Supple Figure S2. Percentage change in body weight of experimental mouse groups over the 12-week treatment period (average amounts of actual body weight at start-point, *Lmna*$^{WT/WT}$: 37.85g and *Lmna*$^{G609G/WT}$: 23.66g). Treatment with Progerinin delayed the body weight loss in *Lmna*$^{G609G/WT}$ mice (*Lmna*$^{WT/WT}$: *n*=11; *Lmna*$^{G609G/WT}$ Vehicle: *n*=8; *Lmna*$^{G609G/WT}$ Treated: *n*=8). Statistical analysis was performed using two-way ANOVA followed by Fisher's LSD test, *p<0.05, **p<0.01, ***p<0.001, ****p<0.0001, and ns: not significant.

Supple Figure S3.

A

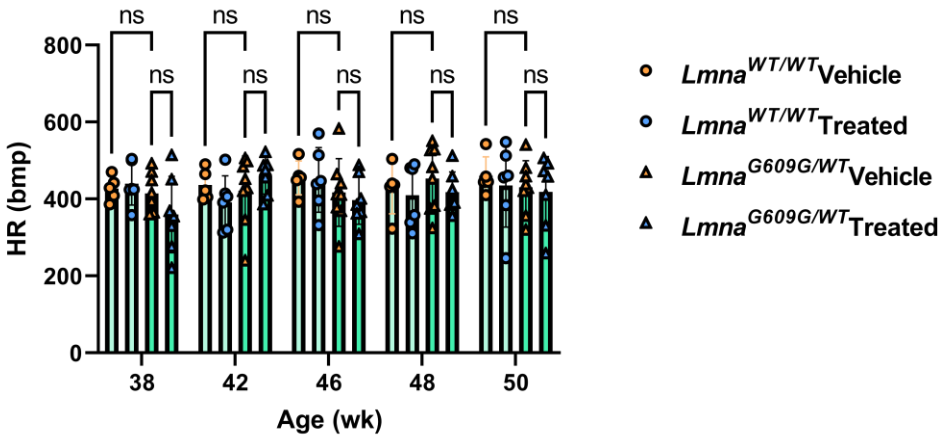

B

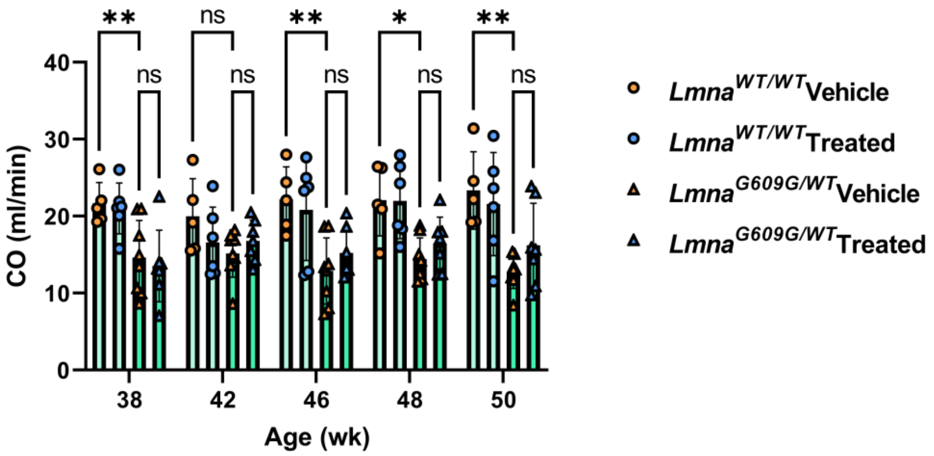

Supple Figure S3. Cardiac parameters of heart rate (HR; (A)); and cardiac output (CO; (B)) measured at 38, 42, 46, 48, and 50 weeks of age in the vehicle and Progerinin-treated mice (*Lmna*$^{WT/WT}$ Vehicle: *n*=5; *Lmna*$^{WT/WT}$ Treated: *n*=6; *Lmna*$^{G609G/WT}$ Vehicle: *n*=8; *Lmna*$^{G609G/WT}$ Treated: *n*=7). Statistical analysis was performed using two-way ANOVA followed by Fisher's LSD test, *p<0.05, **p<0.01, and ns: not significant.

Supple Figure S4.

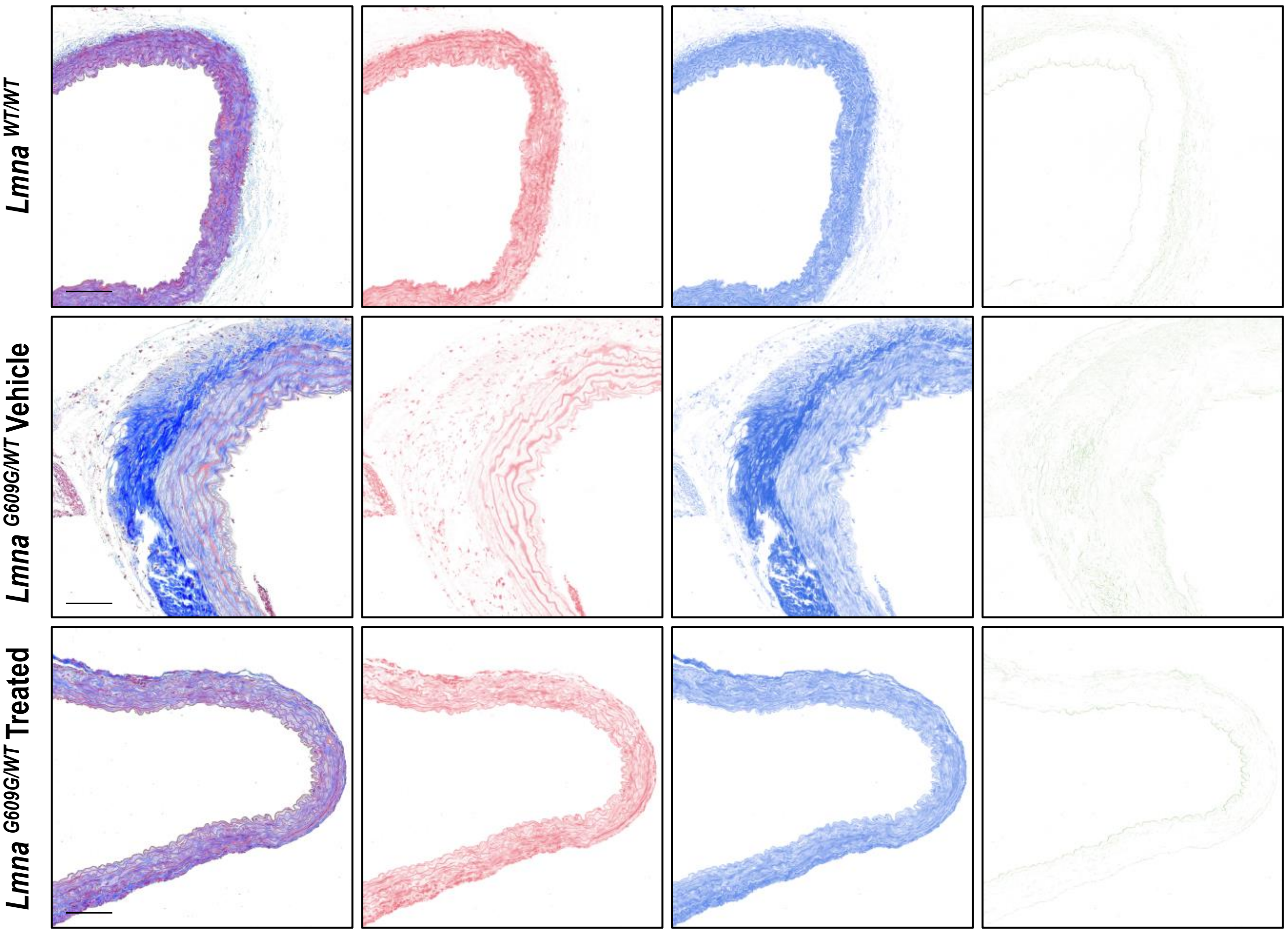

Supple Figure S4. Aortic fibrosis in wild-type mice and *Lmna*$^{G609G/WT}$ mice. Masson's trichrome staining of wild-type mice, vehicle *Lmna*$^{G609G/WT}$ mice, and treated *Lmna*$^{G609G/WT}$ mice (*Lmna*$^{WT/WT}$: *n*=11; *Lmna*$^{G609G/WT}$ Vehicle: *n*=11; *Lmna*$^{G609G/WT}$ Treated: *n*=11). Images of aortic lesions processed by color deconvolution. The scale bar represents 100 µm.

Supple Figure S5.

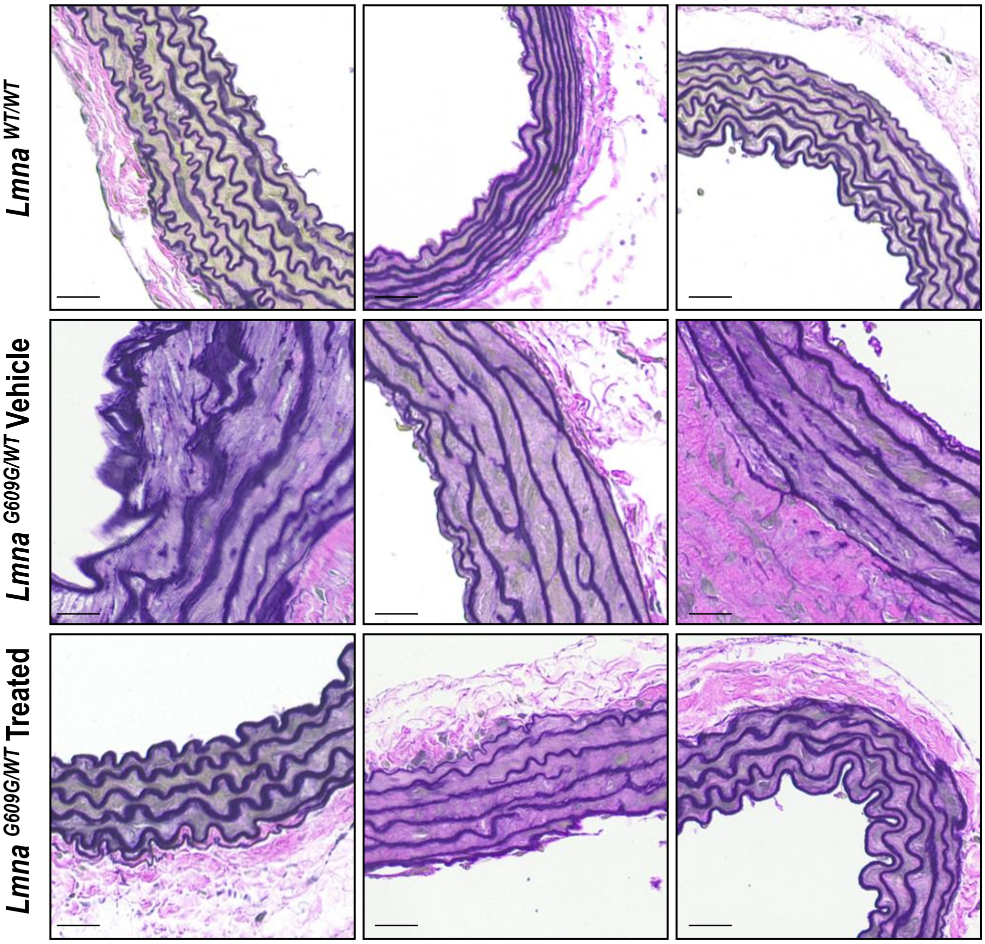

Supple Figure S5. Analysis of elastic fiber of aorta in wild-type mice and *Lmna*$^{G609G/WT}$ mice. Verhoeff-Van Gieson staining of wild-type mice, vehicle *Lmna*$^{G609G/WT}$ mice, and treated *Lmna*$^{G609G/WT}$ mice (*Lmna*$^{WT/WT}$: *n*=11; *Lmna*$^{G609G/WT}$ Vehicle: *n*=11; *Lmna*$^{G609G/WT}$ Treated: *n*=11). The scale bar represents 25 µm.

Supple Figure S6.

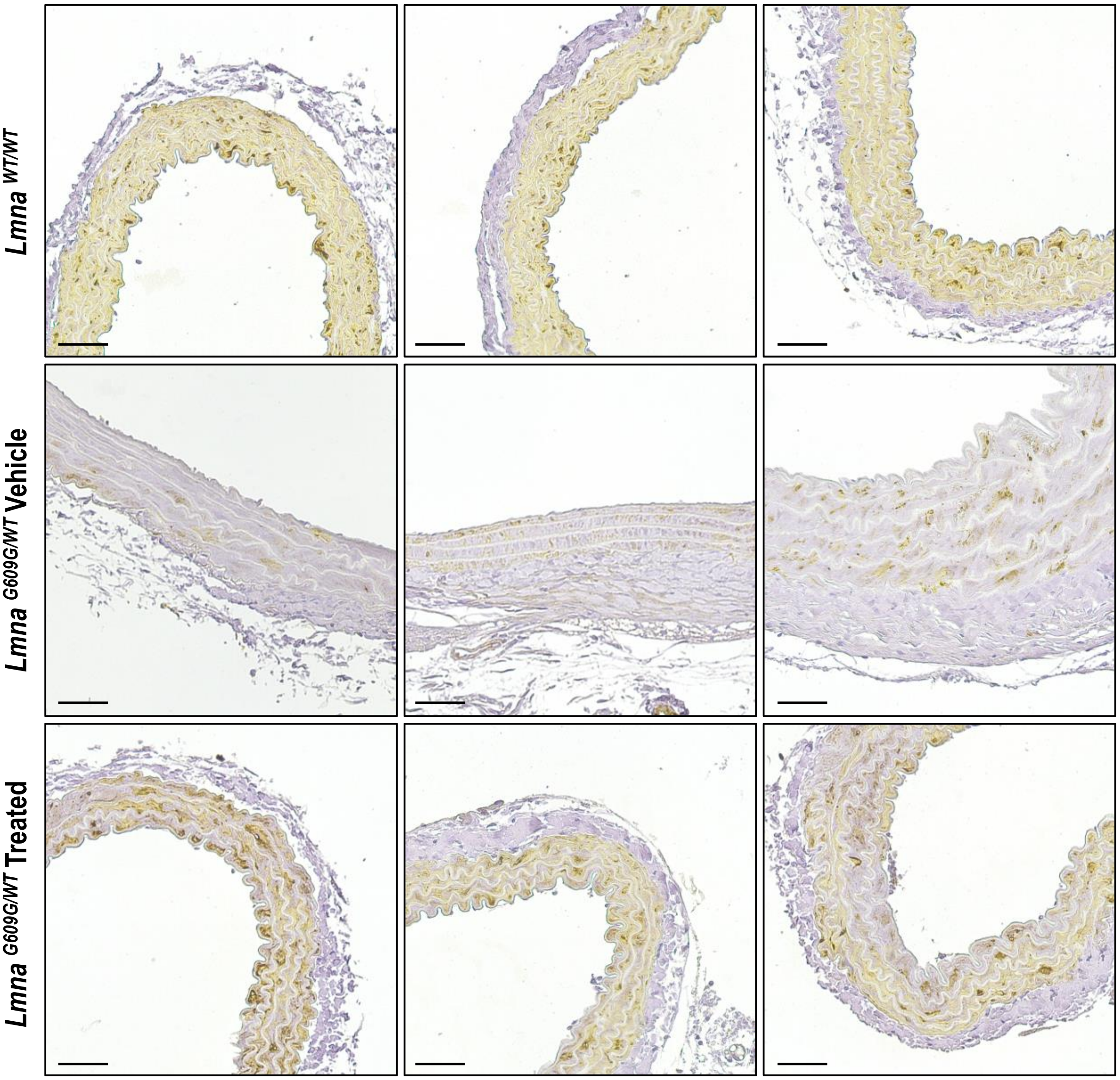

Supple Figure S6. Composition of smooth muscle cells in wild-type mice and *Lmna*$^{G609G/WT}$ mice. Immunohistochemistry (IHC) of aorta stained with an anti-αSMA antibody (*Lmna*$^{WT/WT}$: *n*=11; *Lmna*$^{G609G/WT}$ Vehicle: *n*=11; *Lmna*$^{G609G/WT}$ Treated: *n*=11). The scale bar represents 50 µm.

Supple Figure S7.

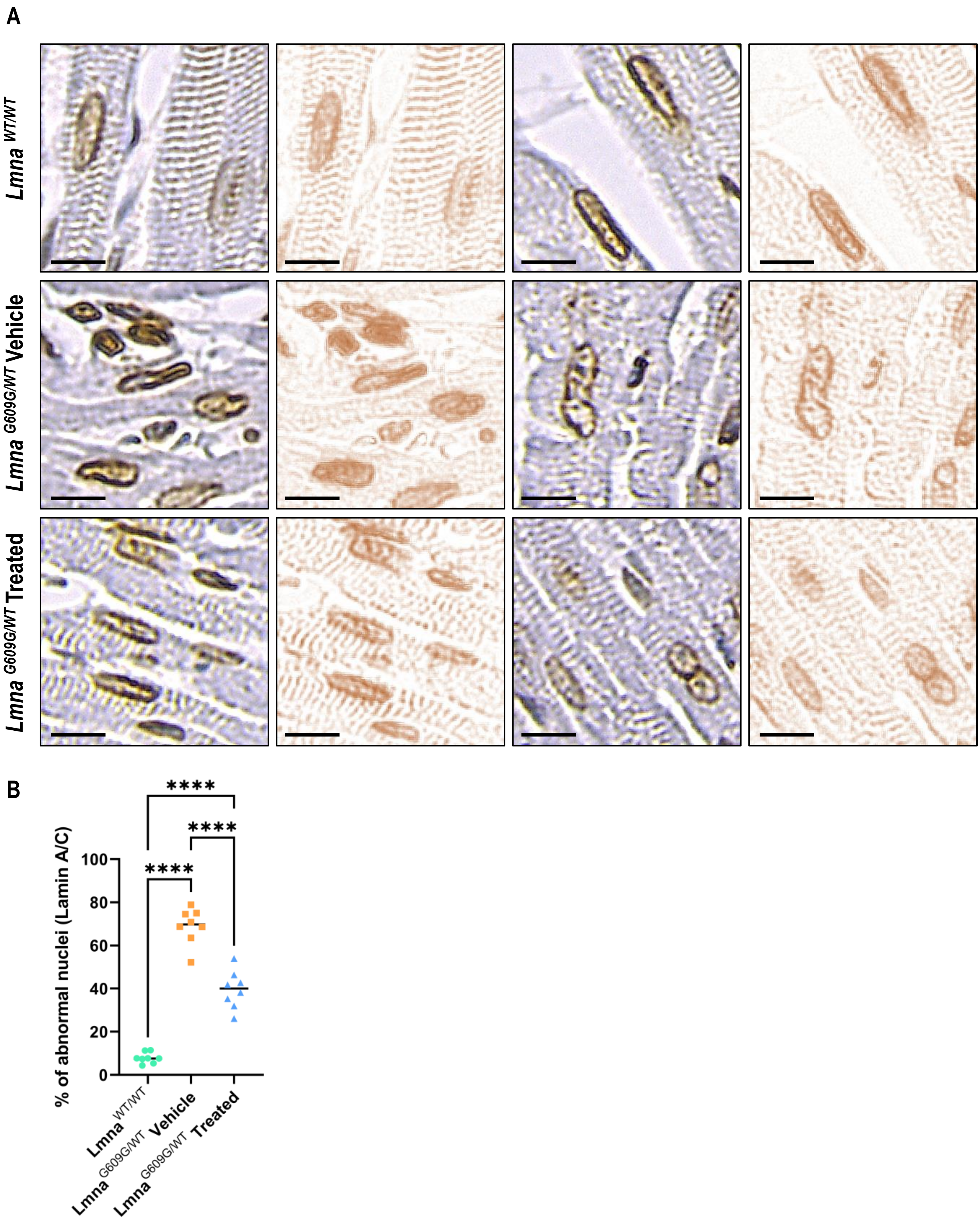

Supple Figure S7. Abnormal nuclei in heart tissue are restored by Progerinin administration. (A) Immunohistochemistry (IHC) of heart tissue sections stained with an anti-lamin A/C antibody (*Lmna*$^{WT/WT}$: *n*=8; *Lmna*$^{G609G/WT}$ Vehicle: *n*=8; *Lmna*$^{G609G/WT}$ Treated: *n*=8). Images of heart lesions processed by color deconvolution. The scale bar represents 10 μm. (B) Quantification of nuclear shape abnormalities in heart tissue. The bar graph shows the percentage of abnormal nuclei within a total of 300 nuclei. Treatment with Progerinin showed a significant reduction (****p<0.0001 by one-way ANOVA followed by Tukey's test) compared with the vehicle *Lmna*$^{G609G/WT}$ mouse group.

Supple Figure S8.

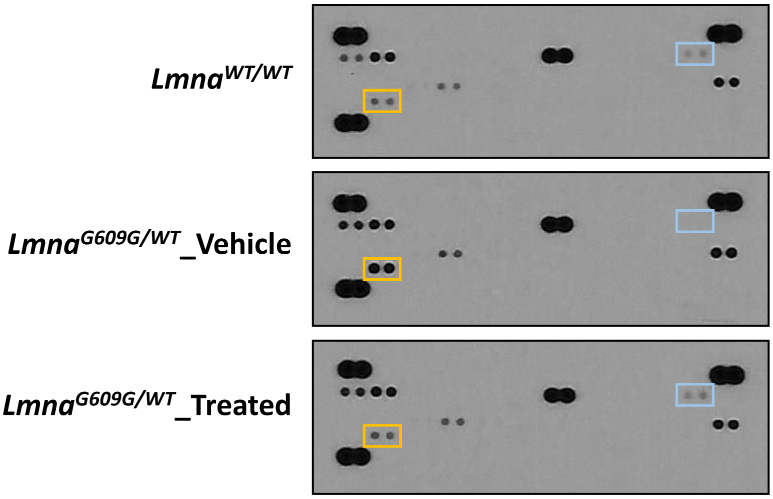

Supple Figure S8. Examples of Mouse Cytokine Antibody Array blots probed with the isolated blood serum samples. Samples from wild-type *Lmna*$^{WT/WT}$ mice were used as a control. Each dot represents immunoreactive staining against respective antibodies. The orange boxes in each membrane indicate the expression of TIMP-1. The blue boxes indicate the expression of IL-1ra.

Table S1.

Table 1. Analysis of cardiac function by echocardiography

Age (wk)	Diet-fed (wk)	Genotypes/Diet	Cardiac function							
			HR (bpm)	SV (ul)	EF (%)	FS (%)	CO (ml/min)	LVPWs (mm)	LVPWd (mm)	DWS (%)
38	0	Lmna$^{WT/WT}$ control	427±29	50.69±4.96	56.06±6.25	29.29±4.30	21.63±2.45	1.24±0.06	0.87±0.07	29.45±8.30
		Lmna$^{WT/WT}$ D011	440±55	48.91±11.61	54.60±5.33	28.21±3.28	21.02±3.30	1.10±0.17	0.82±0.10	26.86±4.44
		Lmna$^{G609G/WT}$ control	415±51	35.35±10.73	54.99±5.83	28.14±3.51	14.63±4.81	1.08±0.13	0.80±0.12	25.98±8.97
		Lmna$^{G609G/WT}$ D011	360±99	37.28±4.71	52.35±4.94	26.55±3.13	13.53±4.66	1.03±0.18	0.71±0.11	30.76±3.23
42	4	Lmna$^{WT/WT}$ control	436±35	45.6±7.64	63.92±4.46	34.44±3.14	20.0±4.38	1.28±0.05	0.85±0.06	33.74±7.43
		Lmna$^{WT/WT}$ D011	391±70	42.08±5.83	61.83±3.93	32.84±2.71	16.58±4.61	1.28±0.09	0.87±0.11	32.45±6.30
		Lmna$^{G609G/WT}$ control	422±90	36.26±4.44	54.83±7.95	28.20±4.91	15.16±3.07	1.05±0.16	0.79±0.16	24.99±4.99
		Lmna$^{G609G/WT}$ D011	461±48	36.51±4.88	59.53±10.17	31.51±7.26	16.80±2.69	1.07±0.17	0.78±0.15	26.43±8.59
46	8	Lmna$^{WT/WT}$ control	455±39	48.7±4.71	65.79±5.38	35.97±4.20	22.18±3.30	1.25±0.12	0.89±0.05	28.66±9.46
		Lmna$^{WT/WT}$ D011	450±84	45.80±10.92	69.63±5.29	38.79±4.26	20.82±6.55	1.25±0.13	0.91±0.04	26.73±7.17
		Lmna$^{G609G/WT}$ control	417±80	30.64±6.62	53.32±9.42	27.16±5.86	12,88±4.31	1.02±0.18	0.77±0.13	24.40±7.10
		Lmna$^{G609G/WT}$ D011	397±63	38.25±3.59	64.54±10.99	35.20±8.11	15.23±3.12	1.13±0.20	0.81±0.11	27.58±9.60
48	10	Lmna$^{WT/WT}$ control	427±58	50.53±5.59	68.61±5.95	38.22±4.61	22.07±4.15	1.33±0.14	0.89±0.07	33.19±4.71
		Lmna$^{WT/WT}$ D011	410±82	53.44±4.94	68.11±5.46	37.83±4.32	21.98±4.90	1.24±0.08	0.82±0.10	34.30±6.02
		Lmna$^{G609G/WT}$ control	453±84	32.32±5.63	56.74±6.85	29.27±4.59	14.42±2.77	1.04±0.14	0.74±0.15	28.37±9.60
		Lmna$^{G609G/WT}$ D011	418±53	39.60±6.73	67.31±6.49	36.90±5.15	16.45±3.44	1.18±0.16	0.76±0.11	35.24±4.48
50	12	Lmna$^{WT/WT}$ control	460±44	49.93±6.78	70.94±2.84	39.87±2.41	23.35±4.50	1.30±0.13	0.90±0.12	30.84±5.04
		Lmna$^{WT/WT}$ D011	435±108	49.17±5.22	71.88±6.13	40.82±5.25	21.58±6.99	1.26±0.11	0.82±0.06	34.20±7.83
		Lmna$^{G609G/WT}$ control	431±69	29.99±4.32	50.93±5.31	25.49±3.23	12.79±2.18	1.05±0.16	0.80±0.08	24.14±5.80
		Lmna$^{G609G/WT}$ D011	418±92	38.98±9.15	65.98±8.96	36.05±6.36	16.22±5.46	1.13±0.14	0.75±0.09	32.95±6.44

Table S1. Analysis of cardiac parameters (heart rate (HR; bmp); stroke volume (SV; µl); ejection fraction (EF; %); fractional shortening (FS; %); cardiac output (CO; ml/min); left ventricular posterior wall thickness at end-systole (LVPWs; mm); left ventricular posterior wall thickness at end-diastole (LVPWd; mm); and diastolic wall strain (DWS; %)) by echocardiography.

Original Western Blot images

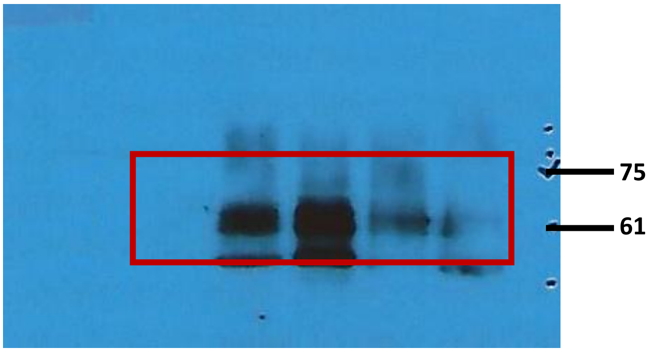

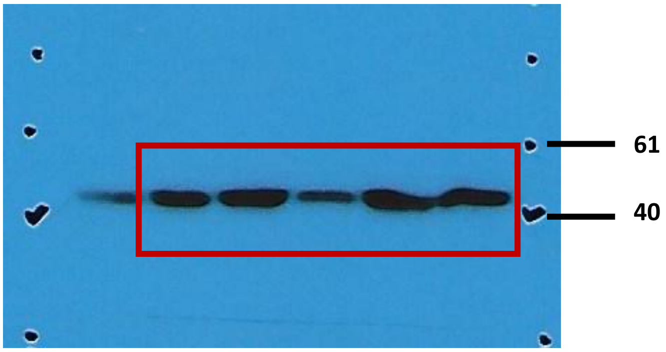

Supplement: Supplementary file 1 [file cells-12-01232-s001.zip › cells-2303432-supplementary.pdf]
